# Exploring the Effect of Ionic Liquid Conformation on the Selective CO_2_ Capture of Supported Ionic Liquid-Phase Adsorbents Based on ZIFs

**DOI:** 10.3390/ma17194829

**Published:** 2024-09-30

**Authors:** Charitomeni M. Veziri, George V. Theodorakopoulos, Konstantinos G. Beltsios, George E. Romanos

**Affiliations:** 1 National Center for Scientific Research “Demokritos”, Institute of Nanoscience and Nanotechnology, 15341 Agia Paraskevi, Greece; g.theodorakopoulos@inn.demokritos.gr (G.V.T.); g.romanos@inn.demokritos.gr (G.E.R.); 2 School of Chemical Engineering, National Technical University of Athens, Zografou Campus, 9 Iroon Polytechniou Street, 15772 Zografou, Greece

**Keywords:** CO_2_ capture, ionic liquids (ILs), supported ionic liquid-phase adsorbents (SILPs), ZIFs, CO_2_/N_2_ selectivity

## Abstract

The CO_2_ adsorption capacity and the CO_2_/N_2_ selectivity of a series of Supported Ionic Liquid-Phase adsorbents (SILPs), including the novel inversely structured SILP “Inverse SILPs”, are thoroughly investigated. ZIF-8, ZIF-69 and ZIF-70 were involved as the solid matrix, while ILs, having tricyanomethanide (TCM) as an anion and alkyl-methylimidazolium of different alkyl chain lengths (C_2_, C_6_, C_8_) as a cation, were used as the liquid constituents of the SILPs. The ultimate target of the work was to ratify a few recently reported cases of enhanced CO_2_ absorptivity in ILs due to their incorporation in ZIFs and to corroborate phenomena of CO_2_/N_2_ selectivity improvements in ZIFs, due to the presence of ILs. This ambiguity originates from the vague assumption that the pores of the ZIF are filled with the IL phase, and the free pore volume of a SILP is almost zero. Yet, through the integration of theoretical predictions with N_2_ porosimetry analysis of an actual sample, it is suggested that a thin layer of IL covered the exterior surface of a ZIF crystal. This layer could act as an impermeable barrier for N_2_, inhibiting the gas molecules from reaching the empty cavities laying underneath the liquid film during porosimetry analysis. This consideration is based on the fact that the solubility of N_2_ in the IL is very low, and the diffusivity at 77 K is negligible. In this context, the observed result reflects an averaged adsorptivity of both the IL phase and the empty pores of the ZIF. Therefore, it is incorrect to attribute the adsorption capacity of the SILP solely to the mass of the IL that ‘hypothetically’ nests inside the pore cavities. In fact, the CO_2_ adsorption capacity of SILPs is always less than the average adsorptivity of an ideal ZIF/IL mixture, where the two phases do not interact. This reduction occurs because some ZIF pores may become inaccessible, particularly when the IL forms a layer on the pore walls, leaving only a small empty core accessible to CO_2_ molecules. Additionally, the IL layer masks the active sites on the ZIF’s pore walls. It should also be noted that the CO_2_/N_2_ selectivity increases only when the ZIF’s pores are completely filled with the IL phase. This is because ILs have a higher CO_2_/N_2_ selectivity compared to the bare ZIF.

## 1. Introduction

Experimental studies have highlighted the potential of metal–organic frameworks (MOFs) in CO_2_ separation applications [1]. In contrast to conventional nanoporous adsorbents, MOFs offer numerous benefits, including heightened adsorption selectivities, which are attributed to the specific interactions between gas molecules and the functional groups within the MOF structure. Furthermore, the expansive surface area of MOFs provides abundant adsorption sites, enhancing gas capture efficiency. The ability to customize MOF properties through synthetic techniques allows for the creation of materials tailored to specific gas separation processes. The primary method for modifying factors like the pore dimensions, surface properties, and structural durability of MOFs is by changing the organic ligands [2]. However, this turns out to be a challenging endeavor due to the restricted combinations of metals and organic ligands capable of forming microporous crystal structures. The post-synthesis functionalization of MOFs, which involves modification of the organic linkers within the MOF structure, is a powerful strategy to impart the particular properties of these materials, making them highly versatile and effective for specific applications [3,4]. A newly developed method for enhancing the gas separation capabilities of an MOF involves the employment of ionic liquids (ILs) [5]. ILs have also been extensively studied as CO_2_ capture systems, thanks to their intrinsic characteristics, such as low vapor pressure, high solubility for CO_2_, nonflammability, and flexibility, in the combination of cation and anion structures like MOFs [6]. It is noteworthy to observe the remarkable advancements as regards the capture and separation of CO_2_ upon the utilization of porous adsorbents based on ILs. Supported Ionic Liquid-Phase adsorbents (SILPs) have gained significant attention in recent years as they combine the desirable characteristics of both ILs and solid supports, offering advantages such as tunable chemical and physical properties, improved stability, and enhanced recyclability [7,8,9]. Their utility extends across a wide array of disciplines, including catalysis, gas separation, electrochemistry, and biomass processing. Supported ILs denote ILs that are confined or immobilized within a solid matrix or support material. This immobilization can be accomplished through diverse techniques, such as physical absorption, encapsulation, or chemical bonding. The combination of ILs and MOFs offers exciting opportunities for the design of advanced materials with tailored properties and synergistic effects for enhanced performance. Typically, the predominant method for preparing supported IL/MOF adsorbents involves impregnation or physical stirring techniques, which promote interactions between a conventional IL and the MOF support, predominantly driven by van der Waals forces or noncovalent bonding [10]. Da Silva et al. pioneered the integration of conventional ILs into MOFs for CO_2_ capture. Their study investigated the impact of incorporating [Bmim][PF6] and [Bmim][Tf2N] into Cu-BTC via dry impregnation on the CO_2_ adsorption capacity. While theoretical calculations suggested that the incorporation of [Bmim][PF6] would enhance CO_2_ adsorption, experimental results revealed a lower adsorption capacity compared to pure Cu-BTC, especially at low pressures. Additionally, high IL loadings (~10 wt.%) led to damage to the Cu-BTC framework, likely due to its narrow pore apertures. Despite this, the composite systems exhibited higher CO_2_ selectivity over N_2_ or CH_4_ compared to neat MOF [11]. Selginel et al. made an intriguing observation regarding the incorporation of [Bmim][BF4] into Cu-BTC. They found that the highest CH_4_ capacity was achieved when the pores were fully occupied by IL, indicating the necessity for high IL loadings without compromising the structural integrity of the MOF. Thus, achieving exceptional gas affinities and separation performances requires careful selection and combination of specific ILs and MOFs [12]. Mohamedali et al. examined two imidazolium-based ILs, specifically [Bmim][Ac] and [Pmim][Tf2N], when combined with Cu-BTC through impregnation. The [Bmim][Ac]/MOF composite demonstrated greater thermal stability, indicating robust interactions between [Bmim][Ac] and the HKUST-1 framework. This, in turn, positively impacted CO_2_ adsorption, as [Bmim][Ac]/MOF exhibited higher CO_2_ capacity compared to [Pmim][Tf2N]/MOF, nearly doubling that of bare HKUST-1 at 0.15 bar. Once again, the choice of anion was highlighted for its importance [13]. Xue and colleagues explored the interaction between various porous organic materials and ILs through molecular simulation. They discovered that electrostatic interactions originating from metal centers, along with the 3D networks, enhance the homogeneous dispersion of ILs into the pores. In their investigation, they found that ZIF-8 exhibits high CO_2_ adsorption capacity and selectivity, not primarily due to unsaturated metal centers but, rather, owing to its specific pore network [14]. In fact, Kinik et al. found stronger interaction between [Bmim][PF6] and ZIF-8, which enhanced both CO_2_ adsorption capacity and separation efficiency for CO_2_/CH_4_ and CO_2_/N_2_ pairs in the low-pressure region [15]. Ferreira et al. examined the adsorption capacity of a range of 10 ionic liquids (ILs) from the [C_n_mim][NTf2] family when combined with ZIF-8. All composite materials exhibit lower adsorption capacity compared to pristine ZIF-8 in both low- and high-pressure regions, with the exception of ZIF-8 supporting [Emim][Ac], which shows a slight increase in the CO_2_ adsorption capacity at 0.5 bar due to the strong interactions between the specific IL and the gas. Moreover, the incorporation of ILs into the structure of an MOF resulted in improved ideal selectivities, especially at low pressures [16]. In a recent study, Ferreira et al. explored a range of alkylammonium-based ILs for the first time. Their findings reaffirmed that composite materials generally have lower adsorption capacities compared to ZIF-8. However, they observed exceptions with two acetate-based ILs in the low-pressure range, showing enhanced CO_2_/N_2_ selectivity values. This highlights how the structures of ILs and their distinct mechanisms of integration into the ZIF-8 matrix affect adsorption capacities. Additionally, they noted for the first time that the size of the cation could impede its gas sorption capacity [17]. Our group has demonstrated the utilization of [Omim][TCM] to fill the interstices among crystals of an as-grown ZIF-69 membrane prepared via a seeded secondary-growth method. The resulting hybrid membrane exhibited markedly superior selectivity compared to as-grown ZIF membranes, while its permeability surpassed that of the bulk IL alone, due to the presence of ZIF channels, which are not covered by IL. Notably, CO_2_ permeated through the intact ZIF pores 20-times faster than N_2_ and 65-times faster through the bulk IL phase [18].

The encapsulation of ILs within the core of an MOF in a shell-like structure represents a relatively novel approach for fabricating ZIF/IL hybrid materials. Pickering emulsions consist of solid particles with the ability to gather at the interface of two immiscible liquid phases. Microcapsules containing ILs have been successfully prepared using silica, polymers, and GO. This approach aims to improve gas diffusivities within ILs and enhance the CO_2_ selectivity of conventional porous adsorbents. The formation of emulsions relies on factors such as the wettability, concentration, and size of the particles. Binks was among the pioneers in realizing that silica nanoparticles with suitable wettability could effectively stabilize emulsions without the need for a polymeric surfactant [19]. In a recent investigation conducted by Wang et al., ionic liquid droplets were incorporated into silica gels with different levels of hydrophilicity, as well as within polymer matrices. Among preparation conditions, employing the sol–gel method with a high IL loading resulted in the highest CO_2_ adsorption capacity. Moreover, the diffusivity values of IL microemulsions were found to be up to two orders of magnitude higher than those of bulk IL [20]. Graphene oxide (GO) has also been employed as the shell material in hybrid microcapsules incorporating IL, resulting in the encapsulated IL showing enhanced CO_2_ adsorption rates compared to its bulk form [21]. Our group has published innovative supported IL-phase systems termed “inverse” SILPs. These systems are developed by the envelopment of micron-sized IL droplets enclosed within silica nanoparticles and produced through a scalable phase inversion process. Our study demonstrates that these inverse SILPs exhibit promising CO_2_/N_2_ separation performance, achieving a selectivity of 20 at absorption equilibrium and enhancing CO_2_ absorption capacity to 1.5–3 mmol g^−1^ at 1 bar and 40 °C. Furthermore, their CO_2_ absorption kinetics surpass those of conventional SILP systems [22].

Unlike conventional Pickering emulsions, MOFs present unique advantages due to their adjustable characteristics [23]. Stabilization of the IL phase in an IL/water system by Ni-BTC was first reported by Ni et al., who proposed a material with quite attractive properties. A novel IL/ZIF-8 core–shell composite structure was prepared by Nie at al. by dissolving [Emim]_2_[IDA] and [Bmim]_2_[IDA] in methanol, followed by the dispersion of ZIF-8 powder into this solution. The [Emim]_2_[IDA] in the form of a shell in this structure yields a material with notable adsorption capacity and CO_2_/CH_4_ selectivity at 0.2 bar. The thickness of the IL shell can be controlled by the mass of the IL in methanol solution [24]. Zeeshan et al. presented a new core–shell configuration of IL/MOF. They deposited [HEmim][DCA] onto ZIF-8. The [HEmim][DCA]/ZIF-8 exhibited approximately 5.7-times greater CO_2_ absorption and 45-times higher CO_2_/CH_4_ selectivity at low pressure compared to ZIF-8. Due to the hydrophilic nature of the selected IL, it formed a shell surrounding ZIF without infiltrating the pores. This configuration proved highly effective for CO_2_ adsorption compared to composites where the IL was loaded into the pores, likely due to the availability of open pores for adsorption [25].

Another method for synthesizing IL/MOF composites arises from the dual potential role of ILs as a solvent and template, with both the cationic and anionic components situated within the cavities. Until now, the literature has been limited concerning ionothermal synthesized ZIF-8 for CO_2_ capture. Ban et al. confined [Bmim][Tf2N] within ZIF-8 cages through in situ ionothermal synthesis and utilized it in the fabrication of polysulfone mixed-matrix membranes. The ZIF-8 composite exhibited a CO_2_/N_2_ selectivity of 100, surpassing that of ZIF-8 and bulk IL, likely due to the confinement effect of IL. In the corresponding mixed-matrix membranes, the positive effect of incorporating ZIF-/IL into the polymer matrix was observed through increased CO_2_/N_2_ and CO_2_/CH_4_ selectivities with increasing pressure, whereas for the pure polymer membranes, the trend was the opposite [26].

The significance of the present study lies in the fact that it is the first to explore all possible pathways for SILP formation using ZIFs of different topologies combined with [TCM]-based ILs with varying alkyl chain lengths. SILPs were prepared upon applications of the following different synthetic methods: (i) simple mixing, (ii) incipient wetness impregnation, (iii) in situ solvothermal reaction, and (iv) nucleation and growth. Inverse SILPs have also been prepared with the inverse-phase method in water, involving various IL/water ratios and consisting of finely dispersed IL droplets of a few micrometers and covered with a porous shell consisting of ZIF crystals. Subsequently, we conducted experiments to investigate the adsorption of CO_2_ and N_2_ under various pressures, at room temperature [27]. In each SILP tested, the findings related to CO_2_ capture and selectivity challenge the prevailing assumption in the literature that ZIF pores are filled with ILs. The morphological and structural features of ZIFs and SILPs were investigated with SEM, XRD, and N_2_ porosimetry. This research study is important in identifying key limitations in current methodologies and offers pathways for improved approaches to ZIF/IL composite design for gas separation applications, particularly in CO_2_ capture.

## 2. Materials and Methods

### 2.1. Synthesis of ZIFs and ZIF/IL Composites

#### 2.1.1. Synthesis of ZIF-8, APTES/ZIF-8, ZIF-69 and ZIF-70

ZIF-8 was synthesized by mixing solutions of 1.50 g Zn(NO_3_)_2_·6H_2_O in 50 mL methanol and 1.67 g 2-methylimidazole in 50 mL methanol. The reaction took place at room temperature for 30 min under stirring. The resulting milky liquid was centrifuged at 5000 rpm for 15 min, and the supernatant was removed. Subsequently, the precipitate was washed three times with methanol, and the resulting powder was dried at 85 °C overnight to obtain the final ZIF-8 crystals. For the synthesis of APTES/ZIF-8, a standard procedure was followed: 200 mg of ZIF-8 was dispersed in 30 mL of toluene, to which 5 mg of amino-silane ((3-Aminopropyl)triethoxysilane) was added. The mixture was heated under reflux at 85 °C for 20 h to facilitate the reaction between the amino-silane and ZIF-8. After cooling, the product was then thoroughly washed with ethanol and dried under vacuum at 85 °C.

ZIF-69 was synthesized by mixing 2 mL of a 0.2 M stock solution of 2-nitroimidazole and 2 mL of a 0.2 M solution of 5-chlorobenzimidazole in a 15 mL vial. To this mixture, 2 mL of a 0.2 M solution of Zn(NO_3_)_2_·6H_2_O was added, and the vial was sealed and heated to 85 °C for 96 h. Afterward, the resulting suspension was poured off, and the yellow ZIF-69 crystals were washed three times with dimethylformamide (DMF), collected by filtration, and air-dried. ZIF-70 was synthesized following the same procedure as ZIF-69, using benzimidazole as the second ligand.

#### 2.1.2. Preparation of ZIF-8/IL SILP via Inverse-Phase Method in Methanol

Here, 60 mg of ZIF-8 powder was dispersed in 40 mL methanol via sonication. To this suspension, 40 mg of [C_6_mim][TCM] was added, followed by mixing of the components at 80 °C until methanol was fully evaporated. It should be noted that this ratio of ZIF to IL masses was chosen based on the bulk density (density defined with respect to the total volume of the material including the pores) of ZIF (0.56 g/cm^3^), resulting in a calculated pore volume of 43%. The aim was to introduce an amount of IL that could completely fill the pores without any excess. The sample is denoted as *inverse* SIL@ZIF-8_*m* (where m is methanol).

#### 2.1.3. Preparation of APTES-ZIF-8/IL SILP via Inverse-Phase Method in Methanol

Here, 60 mg of ZIF-8 powder, previously modified by (3-Aminopropyl)triethoxysilane (APTES), was dispersed in 40 mL methanol via sonication. To this suspension, 40 mg of [C_6_mim][TCM] was added followed by mixing of the components at 80 °C until methanol was fully evaporated. The sample is denoted as *inverse* SIL@APTES-ZIF-8_*m*.

#### 2.1.4. Preparation of ZIF-8/IL SILP via Inverse-Phase Method in Water

Here, 60 mg of ZIF-8 powder was dispersed in 10 mL water via sonication. To this suspension, 40 mg of [C_6_mim][TCM] was added. ZIF nanocrystals tended to form aggregates immediately after introduction of IL into the suspension, which were recovered via water evaporation at 80 °C. The same procedure was repeated by increasing the amount of ZIF-8 relative to IL (60 mg ZIF-8, 15 mg IL). The sample is denoted as *inverse* SIL@ZIF-8_*w*(1) and *inverse* SIL@ZIF-8_*w*(2) (where w is water, (1) is the mass ratio of ZIF-8 to IL of 1.5 and (2) the mass ratio of ZIF-8 to IL of 4).

#### 2.1.5. Preparation of ZIF-8/IL SILP via Incipient Wetness Impregnation

Prior to SILP preparation, all ZIF samples were dried under vacuum to remove moisture and/or any other volatile impurities. For the preparation of ZIF-8/IL sample, 5 mL of methanol was added to 300 mg of [C_6_mim][TCM], and the vial was mixed until the IL was dissolved. The solution was then added to the pre-weighted ZIF-8 material (250 mg) under vacuum, and the solution was mixed overnight in a sealed vial to disperse IL homogeneously into the micropores of ZIF-8. The powder was then recovered by drying at 80 °C until methanol was fully removed. For the preparation of the ZIF-70/IL sample, 10 mL of methanol was added to 700 mg of [C_2_mim][TCM], and the solution was stirred until complete dissolution of the IL. The solution was then added to pre-weighed ZIF-70 material (300 mg) under vacuum, and the mixture was stirred overnight in a sealed vial to achieve homogeneous dispersion of the IL and impregnation into the micropores of ZIF-70. The powder was then recovered by drying at 80 °C until all methanol was removed. As in the case of ZIF-8/IL, the amount of IL used for the ZIF-70/IL sample was in excess compared to the amount that could be entrapped in the pores of ZIF. The absence of inverse SILP formation reinforces the notion that all the IL has penetrated the pores of ZIF, while the excess has formed a thin film around the crystals. The IL used for the development of SILP with ZIF-69 was [C_8_mim][TCM], which had a longer alkyl chain compared to the ionic liquids used with ZIF-8 and ZIF-70. ZIF-69 was impregnated with IL in the same manner as ZIF-8 and ZIF-70. The samples are denoted as SIL@ZIF-8, SIL@ZIF-69, and SIL@ZIF-70.

#### 2.1.6. Preparation of ZIF-8/IL SILP via In Situ Solvothermal Reaction

Ionothermal synthesis was carried out for in situ incorporation of IL into the nanocages of ZIF-8. The reactant Zn(NO_3_)_2_·6H_2_O (1.798 g) and 2-methylimidazole (4 g) were dissolved in approximately 60 mL [C_6_mim][TCM] with the assistance of ultrasonic bath and successively stirred at room temperature for 24 h. Then, the sample was separated using centrifugation and washed with methanol. The sample is denoted as *iono*SIL@ZIF-8.

#### 2.1.7. Nucleation and Growth of ZIF-8 Nanocrystals and Their Conformation as Continuous Shells at the Water/IL Interface

In this method, nucleation and growth of ZIF-8 nanocrystals and their formation of continuous shells take place at the water/IL interface at room temperature. The oil phase was prepared by dissolving 13 mg of zinc acetate dihydrate into 25 mL of deionized (DI) water. Subsequently, 40 mg of IL was added to the above solution, and oil droplets were immediately observed with stirring. Next, the 2-methylimidazole solution in water (375 mg mIm in 6 g H_2_O) was added to the above emulsion, and mixture was stirred for 24 h. Centrifugation was first employed in order to obtain the composite product. However, we observed a separation between bulk IL and ZIF-8 crystals. The composites were finally recovered via water evaporation at 80 °C. The sample is denoted as in situ *inverse* SIL@ZIF-8_*w*.

### 2.2. Characterization of ZIF Supports and SIL@ZIFs

Scanning electron microscopy (SEM) was conducted with a JEOL JSM-7401F field-emission gun (FEG) SEM (Tokyo, Japan), operating under typical conditions of 10 mA emission current and 2 kV operating voltage. X-ray diffraction (XRD) was used to assess crystallinity and structure of ZIFs and SIL@ZIFs, employing a Siemens D500 X-ray diffractometer (Siemens, Erlangen, Germany) configured in a θ/2θ geometry. Nitrogen adsorption isotherms at 77 K were measured using an Autosorb-1 MP porosimeter (Quantachrome Instruments, Boynton Beach, FL, USA). Prior to each measurement, the samples were degassed under a high vacuum of 10^−5^ mbar at 100 °C for 24 h at the instrument’s outgassing stations. Gas sorption experiments were conducted on ZIFs and SIL@ZIFs, using a custom-made low-pressure gravimetric rig equipped with a CI electronic microbalance (CI Precision, Salisbury, UK), as described in a previous study. For these experiments, small amounts of sample (~42 mg) were placed in the weighing pan and outgassed at 100 °C under vacuum (10^−3^ mbar). The adsorption isotherms for CO_2_ and N_2_ were then measured at 35 °C.

## 3. Results

### 3.1. Morphological and Textural Features of the Resulting ZIF-8/IL SILPs

**Figure 1:**
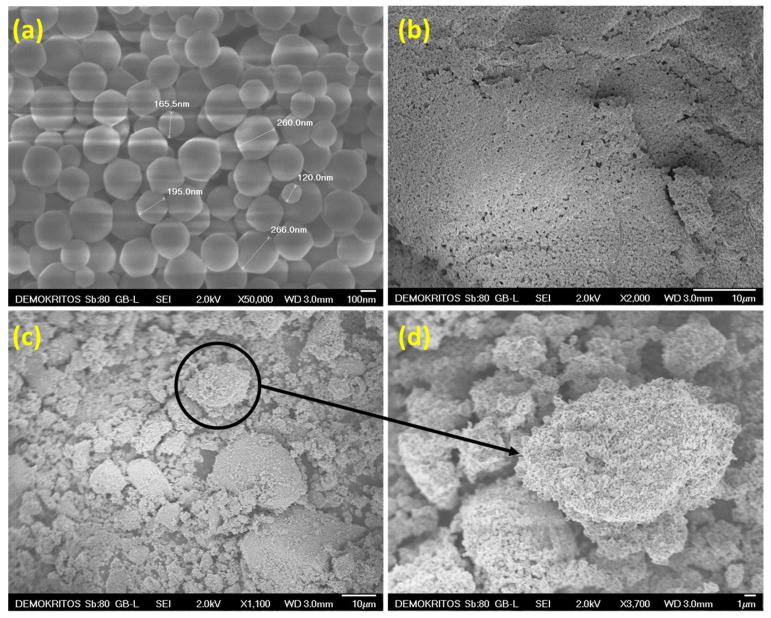
SEM images of: (**a**,**b**) *inverse* SIL@ZIF-8_*m*; and (**c**,**d**) *inverse* SIL@APTES-ZIF-8_*m* prepared in methanol.

Figure 1 contains SEM images of the ZIF/IL systems prepared in methanol. The SEM analysis revealed that the *inverse* SIL@ZIF-8_*m* system did not organize into IL microdroplets coated with ZIF crystals. Upon the functionalization of ZIF-8 with APTES, the *inverse* SIL@APTES-ZIF-8_*m* system organized into IL microdroplets coated with ZIF crystals, as evidenced by the SEM analysis, indicating that phase inversion was achieved. The microdroplets of the IL covered with ZIF crystals are discrete. Here, it should be emphasized that since the quantity of IL used for the SILP formation was sufficient to marginally fill the porous structure of ZIF, the formation of microdroplets of the IL covered with crystals constitutes a first indication that the IL has not entered the pores of ZIF. Conversely, in the case of the sample *inverse* SIL@ZIF-8_*m*, it is uncertain whether the phase of the IL has entered the pores or has created a thin layer around the crystals. Also, while the SILP development conditions were similar for both samples, *inverse* SIL@APTES-ZIF-8_*m* and *inverse* SIL@ZIF-8_*m*, in terms of the solvent and the mass ratio of the IL and solid, the only difference that possibly led to the phase inversion in the case of *inverse* SIL@APTES-ZIF-8_*m* was the coating of the surface of ZIF-8 with APTES; the latter coating significantly altered the surface chemistry and, thus, potentially, the hydrophilicity of the crystals. Whether such inverse SILP phases will form depends on the methanol/IL ratio, the size and content of the nanocrystals in the composite, as well as the polarity of the solvent and the hydrophilicity of the nanocrystals, with the latter possibly being the only difference between the two samples.

**Figure 2:**
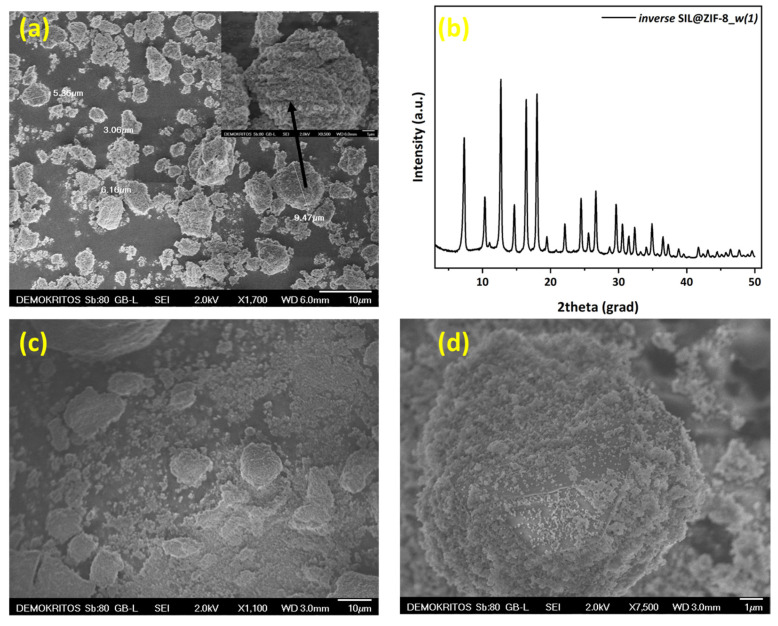
(**a**) SEM image and (**b**) XRD pattern of *inverse* SIL@ZIF-8_*w*(1); (**c**,**d**) SEM images of *inverse* SIL@ZIF-8_*w*(2) prepared in water.

In the case of the ZIF-8/IL SILP prepared in H_2_O, namely *inverse* SIL@ZIF-8_*w*, the ZIF nanocrystals tend to aggregate immediately after the introduction of the IL into the composite recovered via water evaporation at 80 °C. Here, we significantly altered the polarity of the solvent used (compared to the *inverse* SIL@ZIF-8_*m*) by using water instead of methanol. From the SEM micrograph presented in Figure 2a, it is evident that this change also resulted in the successful development of the inverse SILP. Additionally, X-ray diffraction analysis indicates that water did not alter the structure of the ZIF. It is important to emphasize that the quantity of nanoparticles is a critical parameter that determines whether the inversion phase will occur during the evaporation of the solvent to form the inverse SILP. Despite the significant increase in the ZIF/IL ratio of *inverse* SIL@ZIF-8_*w*(2) compared to the *inverse* SIL@ZIF-8_*w*(1), the formation of the inverse SILP phase was achieved once again. This suggests that using a highly polar solvent widens the range of ZIF/IL ratios that can lead to the achievement of phase inversion.

**Figure 3:**
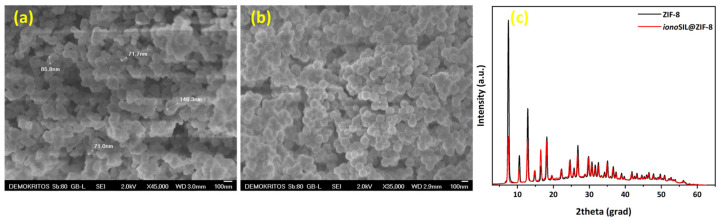
(**a**) SEM image of SIL@ZIF-8 prepared by impregnation under vacuum; (**b**) SEM image and (**c**) XRD pattern of *iono*SIL@ZIF-8 synthesized ionothermally.

In the case of ZIF-8 impregnated under vacuum, a morphology resembling an inverse SILP did not emerge, as shown in the SEM micrograph of Figure 3a. Compared to other SILP materials developed, here, the quantity of IL used was in excess compared to the amount that could be trapped within the pores of the ZIF. The absence of reverse SILP formation supports the assumption that all the IL has penetrated the pores of the ZIF, with any excess IL forming a thin membrane around the crystals. ZIF-70 has much larger pores than ZIF-8 and a comparable specific surface area. The characteristic sizes for the pores of ZIF, d_a_ and d_p_, where d_a_ is the diameter of the largest sphere that can pass through the pores and d_p_ is the diameter of the largest sphere that can fit inside the cell without contacting the lattice atoms, are 3.4 Å and 11.6 Å for ZIF-8 and 13.1 Å and 15.9 Å for ZIF-70, respectively. Furthermore, the IL used for SILP development with ZIF-70 was [C_2_mim][TCM], having a much smaller alkyl chain than the IL used in the case of ZIF-8. Our goal was to determine if the relationship between the molecular size of the IL and the pore size of the ZIF is a significant factor in determining the accessibility of the IL to the intracrystalline channels. As in the case of SIL@ZIF-8, the quantity of IL used for the SIL@ZIF-70 sample was in excess compared to the amount that could be trapped within the pores of the ZIF. The absence of inverse SILP formation reinforces the hypothesis that all the IL has penetrated the pores of the ZIF, while the excess has formed a thin membrane around the crystals. ZIF-69 has approximately the same pore size as ZIF-8, but a much smaller specific surface area. The characteristic sizes for the pores of ZIF, d_a_ and d_p_, where d_a_ is the diameter of the largest sphere that can pass through the pores and d_p_ is the diameter of the largest sphere that can fit inside the cell without contacting the lattice atoms, are 3.4 and 11.6 Å for ZIF-8 and 4.4 and 7.8 Å for ZIF-69, respectively. The IL used for SILP development with ZIF-69 was [C_8_mim][TCM], meaning it had a larger alkyl chain than the ILs used with ZIF-8 and ZIF-70. Once again, our goal was to determine if the relationship between the molecular size of the IL and the pore size of the ZIF is critical for the accessibility of the IL to the intracrystalline channels. In the case of ZIF-69, we worked at the other end, using a large IL and a ZIF with significantly smaller pores.

The SEM image in Figure 3b corresponds to ionothermally synthesized ZIF-8, showing no formation of an inverse SILP phase using this specific SILP formation method. Additionally, the XRD diagrams displayed Bragg reflections corresponding to ZIF-8, indicating that the ionothermal synthesis process successfully produced material with the ZIF-8 structure.

**Figure 4:**
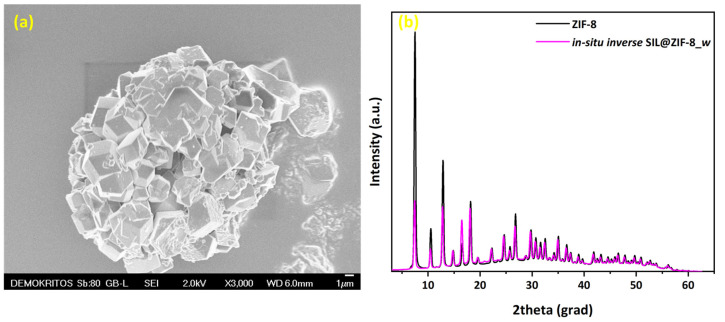
(**a**) SEM image and (**b**) XRD pattern of in situ *inverse* SIL@ZIF-8_*w* synthesized at the water/IL interface.

Regarding the ZIF-8 formation at the IL/water interface, the SEM image in Figure 4a revealed that the in situ *inverse* SIL@ZIF-8_*w* also developed as an inverse SILP. However, the IL droplets were much larger than in previous SILP systems and were covered by significantly larger ZIF crystals. Furthermore, XRD analysis confirmed that the ZIF-8 maintained its structural characteristics in this case as well.

**Figure 5:**
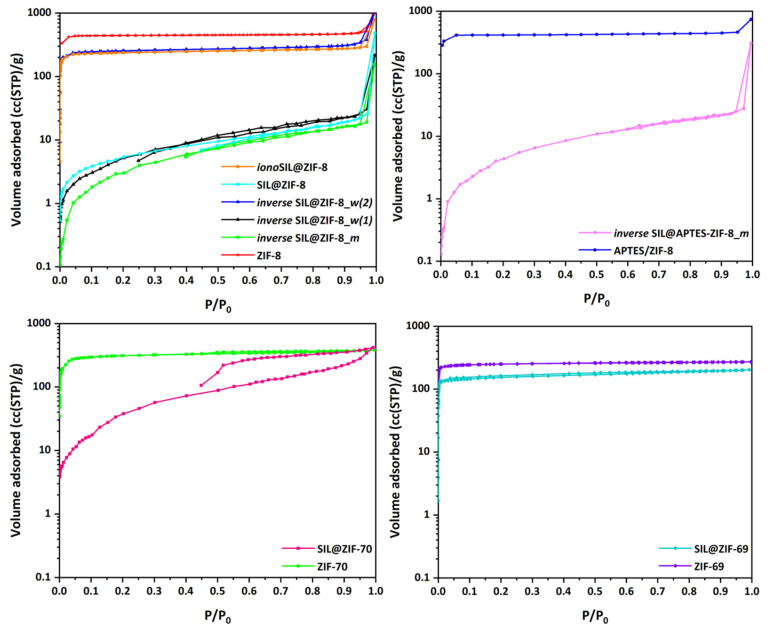
Comparative diagrams of N_2_ adsorption–desorption isotherm curves at 77 K. The comparison is made between all SILPs developed and the corresponding ZIF supports used for their development.

Figure 5 comprises the nitrogen adsorption–desorption isotherm curves at 77 K of all SILP samples developed in this work in comparison to those of the solid substrates (ZIFs) applied for their development. What becomes apparent is that in all SILPs formed with the application of ZIF-8 as a solid substrate, the pore volume of ZIF was almost nullified, except for two cases, i.e., *iono*SIL@ZIF-8 developed through the ionothermal process via the in situ synthesis of ZIF, using the ionic liquid itself as a solvent, and *inverse* SIL@ZIF-8_*w*(2), where the phase inversion is attempted with water as a solvent, but with a high ratio of ZIF mass to the IL. In the first case, in view of the employment of several washes with methanol, during the final stage of SILP recovery, it is possible that the trapped IL in the pores was removed through its dissolution in methanol. In the second case of *inverse* SIL@ZIF-8_*w*(2), a reasonable inference prevails, where the IL is not sufficient either to fully occupy or to fill all the pores of ZIF 8, an assumption supported by the formation of the ‘inverse SILP’, which implies that a large part of the initial quantity of IL has dispersed into microdroplets trapped between crystals, yet outside the intracrystalline pores. In the case of *inverse* SIL@APTES-ZIF-8_*m*, an identical behavior was observed as that of *inverse* SIL@ZIF-8_*m* developed in exactly the same way using unmodified ZIF-8. This is a first indication that the surface chemistry of ZIF is not one of the decisive factors that could control whether the IL will eventually manage to penetrate into the pores of the substrate.

A notable difference in the form of the N_2_ adsorption–desorption isotherm curve for the SILP is observed when using ZIF-70 as the solid substrate. This significant distinction compared to the other SILPs is that, in the case of SIL@ZIF-70, the pore volume has not been diminished, and, furthermore, there is a pronounced hysteresis loop in the desorption branch. The hysteresis phenomena are related to the presence of constrictions at the ends of the pores. Because ZIF-70 has the largest pore size compared to the other two ZIFs and the size of pore aperture, d_a_, is smaller but very close to the size of the pore cavity (d_a_ = 13.1 Å and d_p_ = 15.9 Å), it can be considered that the IL forms a monomolecular layer on the pore walls, and, therefore, the hysteresis is related to further narrowing of the apertures due to the adsorption of the IL. Finally, SIL@ZIF-69 demonstrates the most subtle reduction in pore volume amongst all SILPs developed.

In the next section, the results of nitrogen porosimetry are combined with the results of CO_2_ and N_2_ adsorption of the pure components from which each SILP is composed. Considering the mass ratio between the ZIF and the IL, we formulate an equation predicting the CO_2_ and N_2_ adsorption isotherms of the SILP. Comparing the predicted isotherm with the actual one leads us to valid conclusions about whether the liquid phase of the IL has been introduced into the intracrystalline channels of the ZIF.

### 3.2. Results of CO_2_ and N_2_ Adsorption

The experiments for the adsorption of the two gases (CO_2_, N_2_) were conducted both on the pure components from which the SILPs were developed and on the SILPs themselves. The pure ZIFs were ZIF-8, ZIF-69, ZIF-70, and APTES/ZIF-8, while the pure ILs were [C_2_mim][TCM], [C_6_mim][TCM], and [C_8_mim][TCM]. The equation formulated and used to predict the isothermal adsorption of carbon dioxide and nitrogen in SILPs was of the form:

(1) $$Q_{T}\left(P\right)=y_{ZIF}\times \frac{{TPV}_{SILP}}{{TPV}_{ZIF}}\times Q_{T,ZIF}\left(P\right)+y_{IL}\times Q_{T,IL}\left(P\right)$$

where $Q_{T}\left(P\right)$ (mmol/g) is the predicted absorbed quantity of CO_2_ or N_2_ on SILP, $Q_{T,ZIF}\left(P\right)$ (mmol/g) is the experimental absorbed quantity of CO_2_ or N_2_ on ZIF, $y_{ZIF}$ is the mass fraction of ZIF in SILP, $y_{IL}$ is the mass fraction of IL in SILP, $\frac{{TPV}_{SILP}}{{TPV}_{ZIF}}$ is the fraction of SILP pore volume that remains open, and $Q_{T,IL}\left(P\right)$ (mmol/g) is the experimental adsorbed quantity of CO_2_ in the IL.

#### 3.2.1. CO_2_ Adsorption in ZIFs

**Figure 6:**
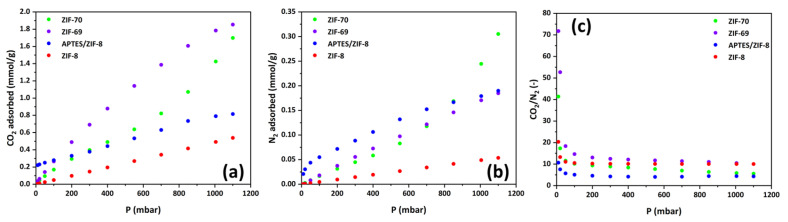
(**a**) CO_2_ and (**b**) N_2_ adsorption isotherms at 35 °C and pressures up to 1 bar of all ZIFs utilized for the development of SILPs; (**c**) corresponding CO_2_/N_2_ selectivities. All experiments were conducted at 35 °C except for the experiment on APTES ZIF 8, which was conducted at 30 °C.

From the results of CO_2_ and N_2_ adsorption on the ZIFs, which are presented in Figure 6 and Table 1, it becomes apparent that ZIF-8, which, as demonstrated by N_2_ porosimetry, has the highest specific surface area and pore volume among the ZIFs used in this study, exhibits the lowest adsorption capacity towards CO_2_. Additionally, ZIF-69, which is characterized by the weakest porous network among the ZIFs, shows the strongest adsorption capacity for carbon dioxide and, simultaneously, the highest CO_2_/N_2_ selectivity. This suggests that, in addition to the porous structure, the surface chemistry of the pores and, thus, the organic linkers used in the synthesis of each ZIF are crucial for the adsorption capacity of CO_2_.

Indeed, in the case of ZIF-69, acid–base Lewis–Lewis interactions between CO_2_ and the pore walls are developed. Recent theoretical studies have confirmed that CO_2_ molecules are adsorbed on ZIF-69 through (a) strong Lewis acid–base interactions between CO_2_ and the nIM (nitroimidazole) linkers, with the electron-deficient carbon atom of CO_2_ acting as a Lewis acid and the two oxygen atoms of the nitro group acting as Lewis bases; (b) weaker hydrogen bond-type interactions (CH…O) between the oxygen atoms of CO_2_ and the hydrogen atoms in the phenyl ring of cbIm (chlorobenzylimidazole), whose strength is increased by the presence of chlorine atoms that attract electrons from the bIm linkages. The first mechanism occurs more prominently in the smaller pores of ZIF-69, and due to its strength, saturation of these active sites occurs at low pressures. The above explains why ZIF-69 leads to exceedingly high CO_2_/N_2_ selectivity compared with other ZIFs at low pressures, while at higher pressures, its selectivity converges to that of the other ZIFs [28,29]. Additionally, from the comparison of CO_2_ adsorption results in ZIF-8 and APTES/ZIF-8, a paradoxical conclusion is drawn that, while modification of ZIF-8 with amino groups significantly enhances its adsorption capacity for CO_2_ at low pressures (having the highest adsorption capacity among all tested ZIFs up to 50 mbar), its selectivity remains lower than that of pure ZIF-8. The main reason proposed for this is that, alongside the enhancement of CO_2_ adsorption due to the amino groups of silane, there is also a significant enhancement in N_2_ adsorption, partly due to the lower temperature at which the experiments with APTES/ZIF-8 were conducted (30 °C compared to 35 °C for the other ZIFs), as well as due to the increased surface roughness of APTES ZIF-8 caused by the attachment of aminosilane groups, creating additional active sites for the physical adsorption of nitrogen.

**Figure 7:**
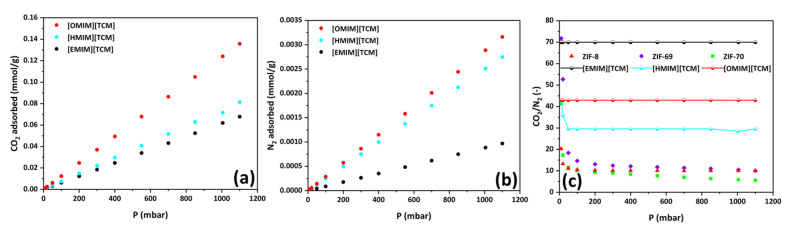
(**a**) CO_2_ and (**b**) N_2_ adsorption isotherms of ILs; (**c**) CO_2_/N_2_ selectivity of the ILs compared to that of ZIFs.

The adsorptive capacity of ILs for CO_2_ and N_2_ is shown in Figure 7a,b. It is evident that the adsorption capacity follows the order (increases) from the IL with the smallest alkyl chain on the imidazole ring of the cation to the IL with the largest alkyl chain. This result is consistent with the theory of free volume in ILs, in which the space where CO_2_ is absorbed is located between the cation and the anion, and, therefore, it is desirable for the imidazole ring to be modified with a large side chain, which weakens the interactions between the anion and cation, creating a larger free volume. What is very important to emphasize is the significantly lower absorption capacity of ionic liquids towards CO_2_ compared to the adsorption capacity of ZIFs. For example, the best IL, namely [OMIM][TCM], absorbs 0.136 mmol/g CO_2_ at 1100 mbar pressure and 35 °C temperature, whereas the weakest, ZIF (ZIF-8), adsorbs 0.54 mmol/g CO_2_ under the same pressure and temperature conditions. Therefore, the question arises whether the effort to entrap ILs in the pores of ZIFs, a process that has been extensively studied by numerous researchers in recent years, could enhance the adsorptive capacity and selectivity of the solid absorbent. It should also be mentioned that although we are comparing the CO_2_ uptake per unit mass of the adsorbent medium, the result would be the same even if the comparison was made per unit volume. Volumetric comparison is more interesting from a practical perspective as it also relates to the footprint of the process, since the density of both the examined ZIFs and ILs is similar, close to 1 g/cm^3^. The answer comes by examining the curves in Figure 7c. The results show that only in the case of ZIF-69 CO_2_/N_2_, selectivity values which can exceed that of ILS can be achieved, but this occurs at very low pressures below 50 mbar. As previously mentioned, there are quite strong interactions between the pore walls of ZIF-69 and CO_2_, which are either of the LA-LB or hydrogen bonding type. These interactions, developed at specific active sites on the pore surface, are exhausted with a pressure increase, as saturation of the active sites with adsorbed CO_2_ molecules occurs [28,29]. Thus, at pressures greater than 50 mbar, the selectivity of all ZIFs becomes significantly lower than that of ILs, with the latter exhibiting physical adsorption in the entire pressure range.

#### 3.2.2. Comparison of Predicted and Experimental CO_2_ and N_2_ Adsorption Isotherms on SILPs

Based on Equation (1), utilizing adsorption data in pure ZIFs and ILs, and considering the relative mass of the ionic liquid/ZIF and the fraction of volume of ZIF pores remaining open after the addition of the IL, it was possible to predict the adsorption isotherms of CO_2_ and N_2_ in all SILPs. Table 2 presents the constant parameters used in each SILP (mass fraction and fraction of pore volume remaining open) for predicting the adsorption isotherms for CO_2_ and N_2_.

**Figure 8:**
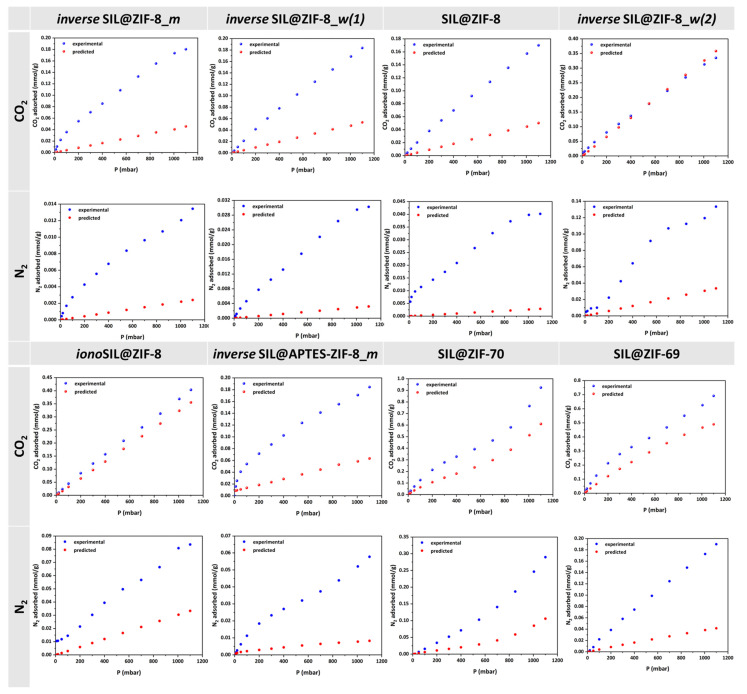
Adsorption isotherms of CO_2_ and N_2_ at 35 °C of all developed SILPs compared to the corresponding isotherms predicted by Equation (1).

As shown in Figure 8, in all cases of the studied SILPs, the predicted adsorption isotherms derived from Equation (1) demonstrate significantly lower storage capacity for CO_2_ and N_2_ compared to the actual storage. This result leads to the conclusion that the reduction in the volume of SILP pores, as observed from N_2_ porosimetry, is deceptive. Actually, in no case of SILP samples does the IL manage to penetrate into the intracrystalline channels of the ZIF but, rather, forms a thin film around the crystals. This film is impermeable to N_2_ molecules under the conditions of N_2_ porosimetry, firstly because nitrogen is only slightly soluble in ILs and, secondly, because the temperature is very low (77 K), resulting in minimized diffusion. On the other hand, when an experiment is conducted at room temperature, diffusion can be measured. CO_2_ and N_2_ molecules penetrate into the thin film of the IL and diffuse towards the pores of the ZIF until they fill them. Therefore, the actual result obtained is the average of the adsorption isotherms of the pure components, weighted by the mass of each component in the SILP. With this methodology, we demonstrated that it is ultimately erroneous to assume that the IL fills the pores of the ZIF and to determine the absorptive capacity of the composite material (SILP) based solely on the theoretical mass of the IL that has infiltrated the pores. In general, the conclusion that emerges from this study is that the creation of a SILP using a ZIF as the solid support and an IL as the liquid phase induces a decrease rather than an increase in the adsorption capacity of the ZIF. However, it is imperative to examine the CO_2_/N_2_ selectivity values of the composite materials in order to more safely evaluate the potential importance of SILPs, for which there is a significant current interest, as regards the CO_2_ capture process.

#### 3.2.3. Selectivity of SILPs

The results presented in Figure 7 pertain to the selectivity of the pure components comprising the SILP and show that while ZIFs exhibit high selectivity values for CO_2_ at low pressures up to 50 mbar, ILs outperform ZIFs in the high-pressure region up to 1000 mbar. This is primarily attributed to the fact that CO_2_ interacts via the carbon atom or oxygen atoms with the functional groups present on the pore walls of the ZIF. These interactions appear as either LA-LB-type or strong hydrogen bonding interactions. When these active sites saturate with adsorbed CO_2_ molecules at low pressures, the interactions weaken, and, subsequently, the channels are filled via physical adsorption. ΙLs, on the other hand, exert physical absorption from low pressures, and, therefore, the selectivity remains stable across the entire pressure range studied.

**Figure 9:**
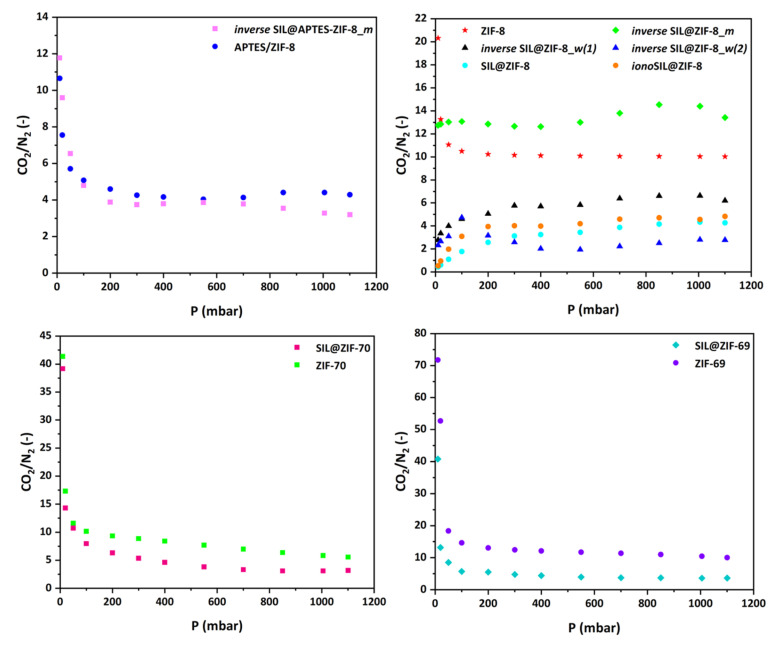
Comparison between the CO_2_/N_2_ selectivity values of ZIFs and the corresponding SILPs developed by various preparation procedures.

The selectivity diagrams depicted in Figure 9 indicate that, in most cases, the CO_2_/N_2_ selectivity values of SILPs decrease and, moreover, exhibit values lower than those found for pure ZIFs. However, this trend is not consistent across all composites. Notably, in two cases, namely *inverse* SIL@APTES-ZIF-8_*m* and *inverse* SIL@ZIF-8_*m*, the incorporation of IL resulted in a positive impact on selectivity values towards CO_2_. Both these samples utilized a similar development approach, wherein an attempt was made to create an inverse SILP using the phase inversion technique with methanol as the polar solvent. A previous study by our group demonstrated that the preparation of an analogous sample led to an enhancement in CO_2_ selectivity [14]. Thus, a preliminary conclusion that might be drawn is that the creation of an inverse SILP adsorbent was achieved only in these two cases.

## 4. Conclusions

This work provides critical insights into the limitations of current methodologies used to investigate IL incorporation in ZIF pores and the extent of pore filling by ILs. The main conclusion that emerges from the present study is that, regardless of the pore size of the ZIF and the size of the side chain on the imidazole ring of IL, filling the ZIF pores with IL was not achieved in any case of SILP formation, despite the possibility of the opposite conclusion based on the methodology that prevails in the current pertinent literature. According to the latter, the wettability capacity of SILP is related to the mass of the ionic liquid that “theoretically” fills the intracrystalline pores of ZIF, but the calculation is flawed as it is based on N_2_ porosimetry results, which show a pseudo-filling of the ZIF pores. Based on the scientific findings of this work relative to the CO_2_ absorption capacity and CO_2_/N_2_ selectivity, we conclude that the involvement of physically absorbing IL in SILP formation with a ZIF as a solid substrate has, in most cases, a negative outcome.

Among the techniques for creating SILPs, the approach that successfully achieved phase inversion involved use of a polar solvent (methanol) to dissolve the IL. During the solvent evaporation from the stirred mixture of ZIF crystals in the IL/methanol solution, inverse SILPs were developed, as concluded by SEM micrographs. Moreover, these samples exhibited higher CO_2_/N_2_ selectivity values, which surpassed those of the ZIF substrates.

Overall, the results of this study so far indicate that the objectives of increasing adsorption capacity and selectivity via SILP formation have not yet been achieved. However, due to the plethora of different structures and surface chemistry of ZIFs and MOFs, as well as the excessively large combination of anion–cation pairs in ILs, we support the ongoing research in this field because of strong potential for the further development of a suitable combination of ZIFs with ILs characterized by a significant performance enhancement.

## Figures and Tables

**Table 1:** Comparison of the morphological characteristics and adsorption capacity of ZIFs samples.

Sample	S_BET_ (m^2^/g)	CO_2_ (mmol/g) @ 1100 mbar	CO_2_/N_2_ Selectivity @ 20 mbar
ZIF-8	1813	0.54	13
ZIF-69	950	1.85	52
ZIF-70	1730	1.69	17

**Table 2:** Mass fraction of ZIF and IL in SILPs, along with the pore volume fraction of open channels in the SILPs samples.

SILPs	$y_{ZIF}$ (-)	$y_{IL}$ (-)	$\frac{{TPV}_{SILP}}{{TPV}_{ZIF}}$ (-)
*inverse* SIL@ZIF-8_*m*	0.6	0.4	0.04
*inverse* SIL@ZIF-8_*w*(1)	0.6	0.4	0.065
*inverse* SIL@APTES-ZIF-8_*m*	0.6	0.4	0.062
SIL@ZIF-8	0.43	0.57	0.054
*iono*SIL@ZIF-8	0.98	0.02	0.625
*inverse* SIL@ZIF-8_*w*(2)	0.75	0.25	0.80
SIL@ZIF-70	0.3	0.7	0.99
SIL@ZIF-69	0.3	0.7	0.73

## Data Availability

Data available on request from the authors.
